# The ‘Radiant Effect’: Recent Sonographic Image-Enhancing Technique and Its Impact on Nuchal Translucency Measurements

**DOI:** 10.3390/jcm13123625

**Published:** 2024-06-20

**Authors:** Arne Bergsch, Jan Degenhardt, Rüdiger Stressig, Heiko Dudwiesus, Oliver Graupner, Jochen Ritgen

**Affiliations:** 1 Praenatal Plus, Centre for Prenatal Diagnostics and Genetics, 50672 Cologne, Germanyj.ritgen@praenatalplus.de (J.R.); 2 Arbeitskreis Ultraschallsysteme, DEGUM, 10117 Berlin, Germany; 3 Department of Gynecology and Obstetrics, RWTH Aachen University, 52062 Aachen, Germany

**Keywords:** nuchal translucency, first trimester screening, image enhancement, aneuploidies, prenatal diagnostics

## Abstract

**Background**: This study assesses the effects of the ‘Radiant’ image enhancement technique on fetal nuchal translucency (NT) measurements during first-trimester sonographic exams. **Methods**: A retrospective analysis of 263 ultrasound images of first-trimester midsagittal sections was conducted. NT measurements were obtained using a semi-automatic tool. Statistical methods were applied to compare NT measurements with and without ‘Radiant’ enhancement. An in vitro setup with predefined line distances provided additional data. **Results**: Incremental increases in NT measurements were observed with varying levels of ‘Radiant’ application: an average increase of 0.19 mm with ‘Radiant min’, 0.24 mm with ‘Radiant mid’, and 0.30 mm with ‘Radiant max.’ The in vitro results supported these findings, showing consistent effects on line thickness and measurement accuracy, with the smallest mean deviation occurring at the ‘Radiant mid’ setting. **Conclusions**: ‘Radiant’ image enhancement leads to significant increases in NT measurements. To avoid systematic biases in clinical assessments, it is advisable to disable ‘Radiant’ during NT measurement procedures. Further studies are necessary to corroborate these findings and to consider updates to the NT reference tables based on this technology.

## 1. Introduction

The relevance of NT assessment as part of the first-trimester screening (FTS) is proven and internationally accepted as a sonographic standard in the detection of major fetal aneuploidies. First introduced in 1992 by Nicolaides et al., nuchal translucency was the initial sonographic parameter that enhanced sensitivity in first-trimester screening for fetal anomalies [1]. Prior to this, the risk assessment for fetal Trisomy 21 primarily depended on maternal age and hormone levels [2]. By incorporating additional sonographic markers (nasal bone, tricuspid valve flow, ductus venosus flow), the detection rate for fetuses with Trisomy 21 could be increased to over 95%. Due to the fundamental importance of accurate risk calculation, optimal image quality is essential for the precise measurement of the NT [3,4].

Early detection via NT measurement allows for strategic planning and timely medical interventions, ensuring comprehensive prenatal care. In essence, NT measurement during the first trimester is indispensable not only for the early detection of chromosomal and structural anomalies but also for comprehensive fetal health assessment and nuanced risk evaluation. This underscores its vital role in enabling informed clinical decisions and tailored prenatal care throughout pregnancy [5,6,7].

Given the rapidly advancing assets of modern ultrasound devices, an improvement in image quality can be noticed over the years. Some imaging techniques have the potential to alter line thickness and measurements of NT. This algorithm changes the appearance of B-mode sonographic images.

The technology uses the B-mode image to create a 3D render based on the brightness of each part of the image. This results in a virtual three-dimensional landscape in the shape of the lines recorded. Then, the device shines a virtual light source onto the 3D relief from the direction of the ultrasound probe. Parts of the relief that are parallel to the surface reflect the virtual light source and are, therefore, recorded as a strong signal. Lateral parts of each line create a ‘slope‘ in the 3D relief and, therefore, reflect the virtual light to the side, making them less likely to reproduce a signal. This creates an effect of lateral contrast enhancement and, therefore, reduces line thickness.

In our estimation, this effect is most noticeable during NT measurements. The aim of this study was to investigate the impact of the ‘Radiant Image Enhancement Technique’ on the measurement of nuchal translucency (NT) and its subsequent clinical relevance.

## 2. Materials and Methods

### Nuchal Translucency with and without ‘Radiant’ Applied

Images of NT measurements obtained from July to October of 2022 (*n* = 263) on Voluson Expert 22 (GE Healthcare, Solingen, Germany, Software Version EC400) were reviewed in the on-device archive, and the ‘Radiant’ image enhancement technique was applied post-exam directly on the device. The gestational age for all examinations was 11 + 0 to 13 + 6 weeks, as this represents the timespan used for FTS. All examinations were conducted by experienced examiners certified according to the quality criteria of the Fetal Medicine Foundation (FMF) London.

For each examination, if multiple NT images were available, the one that most accurately adhered to FMF standards was selected. FMF standards for NT measurements describe methods for obtaining a true midsagittal section. We checked for those standards—correct magnification, visible nose bone, non-visible zygomatic bone, clearly distinguishable thalamus and midbrain, neutral fetal position, and thin nuchal membrane [8]. Adherence to these standards was ensured because all physicians are certified annually by the FMF for first-trimester screening. Image-enhancing modalities, including ‘Harmonic Imaging’, ‘Ultra HD’, Speckle Reduction Imaging (SRI), and Compound Resolution Imaging (CRI), were activated for all cases, as they are standard in our first-trimester scans. Unlike ‘Radiant’, these settings cannot be deactivated after image capture due to their operational method. Maintaining these settings for all cases helps to minimize confounding variations. In the subsequent text, references to ‘Radiant off’ or ‘Native’ indicate that ‘Radiant’ was not used, while ‘Ultra HD’, SRI, and CRI remained active, as previously mentioned.

For each image, NT measurements were initially taken without the ‘Radiant’ enhancement. Subsequently, the same image underwent repeated measurements with ‘Radiant’ applied at the settings ‘min’, ‘mid’, and ‘max’. These measurements were performed using GE’s ‘SonoNT’ tool (GE Healthcare, Solingen, Germany, Software Version EC400), which allows the user to define a rectangular region within the image for standardized NT measurement using the device. This tool was selected due to its proven ability to significantly reduce interobserver variability [9,10].

**Figure 1:**
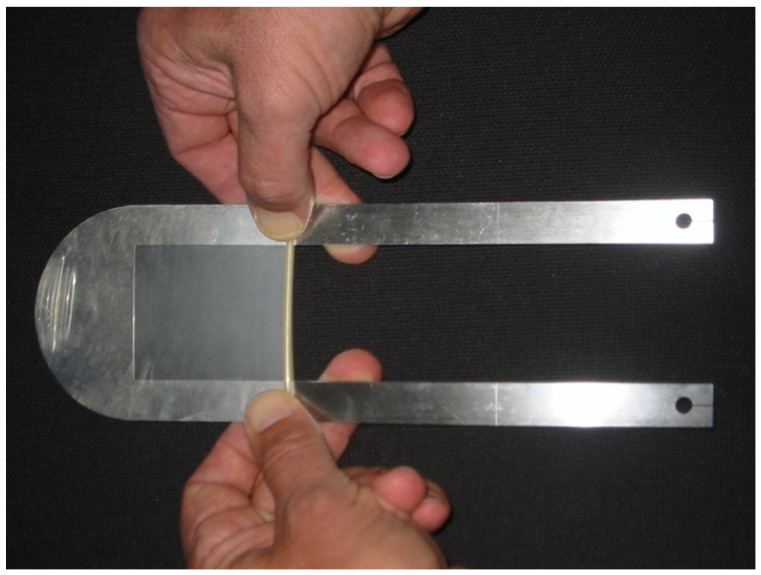
Placement of condom on metal slice with predefined thicknesses.

**Figure 2:**
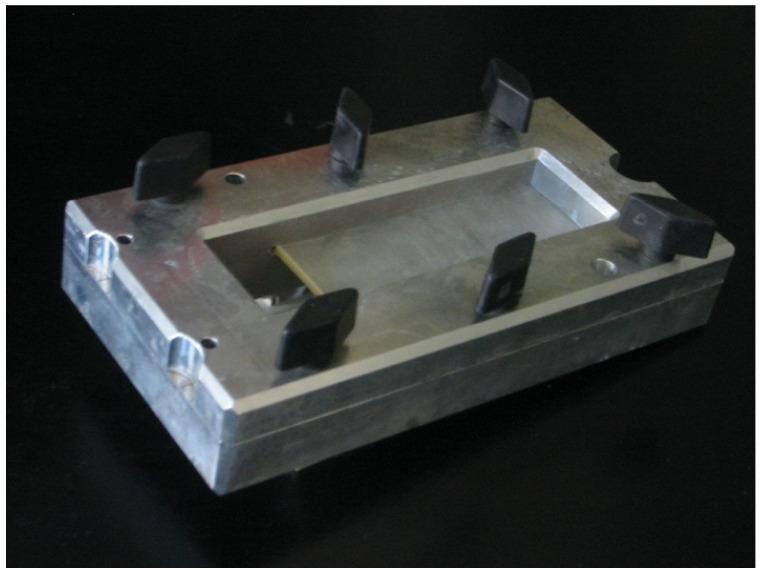
The metal frame locks the spanned condom in place.

**Figure 3:**
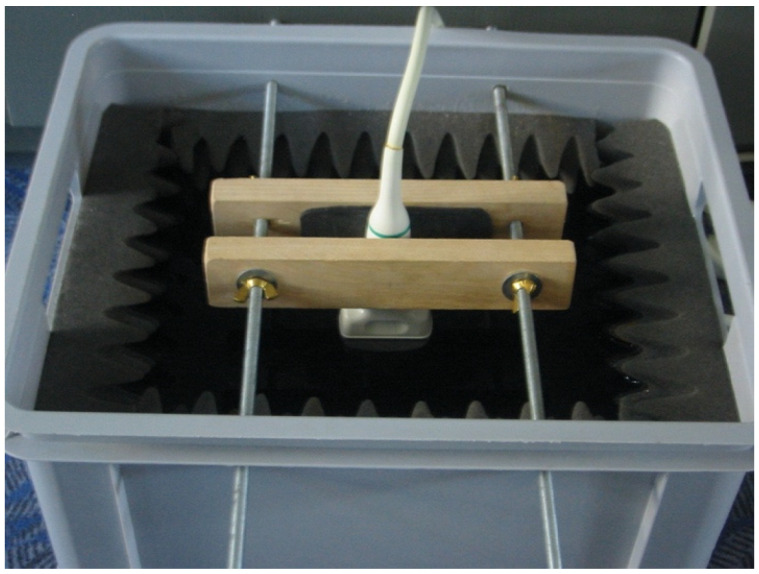
Probe placement in a standardized position. The bucket holds distilled water and is insulated to prevent ultrasonic reverberations. The metal frame is positioned inside. Figure 1, Figure 2 and Figure 3 were provided with kind permission from H. Dudwiesus.

**Figure 4:**
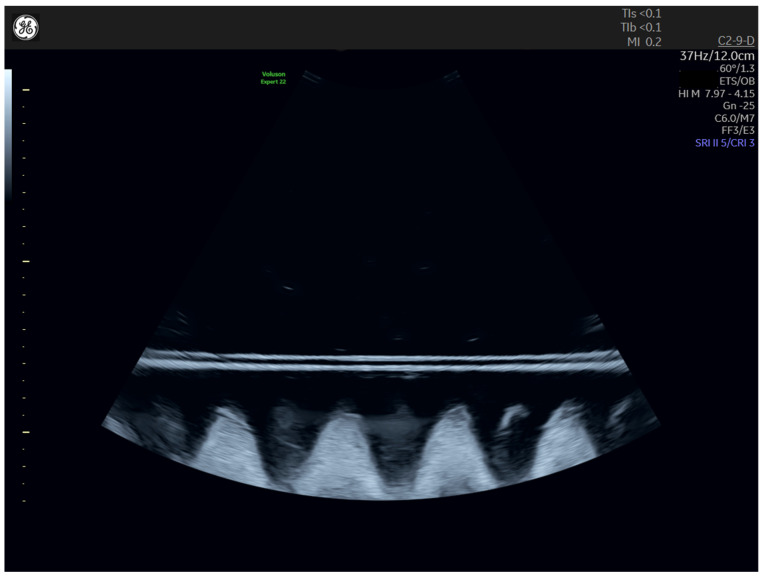
B-mode-image of the in vitro setup. The two horizontal lines are the membranes of a condom, with a predefined distance in between. The cone-like structures below are sound-absorbing foam.

In addition to our patient-based examinations, we conducted an in vitro study using an inorganic object to objectively assess changes in line thickness relative to the actual size of the measured material. For this, we utilized a mechanical device designed to stretch a condom between its membrane walls at a predefined distance. The stretched condom was submerged in distilled water, and the ultrasound probe was positioned underwater, orthogonal to the condom’s membrane. This method replicates the experimental setup used by Heiko Dudwiesus in his prior research on the effects of Harmonic Imaging [11]. The setup is illustrated in the figures (Figure 1, Figure 2, Figure 3 and Figure 4). These measurements were conducted using the same ultrasound machine and probe as in our first-trimester screening to minimize variability.

To establish a setting comparable with clinical data, we measured the distance between the condom membranes under various settings, including the factory preset with ‘Ultra HD’, SRI, and CRI activated, which we regularly use in first-trimester screenings. We also took measurements with ‘Harmonic Imaging’ activated, and with no enhancements (‘Fundamental’), while keeping SRI and CRI activated throughout.

For each setting, we compared ‘Radiant’ with ‘Radiant off’ by measuring the distance between the membranes using the ‘SonoNT’ tool. This examination was conducted with actual distances of 1.0 mm and 2.5 mm between the membranes. Although the operator was aware of the settings being used, the reliance on the automated ‘SonoNT’ tool for measurements obviates the need for operator blinding, as the tool’s design significantly reduces interobserver variability [9,10].

In each examination, details such as gestational age, crown-rump length (CRL), date of the original examination, and the attending physician’s name were documented. In cases with suspected aneuploidy, invasive diagnostics were conducted as part of the clinical procedure (CVS).

The measurements obtained under different ‘Radiant’ settings were analyzed using the t-test for dependent variables, employing IBM SPSS (Version 29.0) software.

For this, the acquired NT values for each step of “Radiant” were compared to the group of NT values measured without “Radiant”. The resulting difference in mean NT was named “ΔNT”. The resulting *p* values of statistical significance are listed in Table 1.

The study was approved by the ethical committee of RWTH University, Aachen, Germany (No. EK 24-039).

## 3. Results

Table 2 shows the mean values of gestational age (GA), crown-rump length (CRL), NT, and maternal age by group, along with their relative frequencies.

### 3.1. Nuchal Translucency with and without ‘Radiant’ Applied

**Figure 5:**
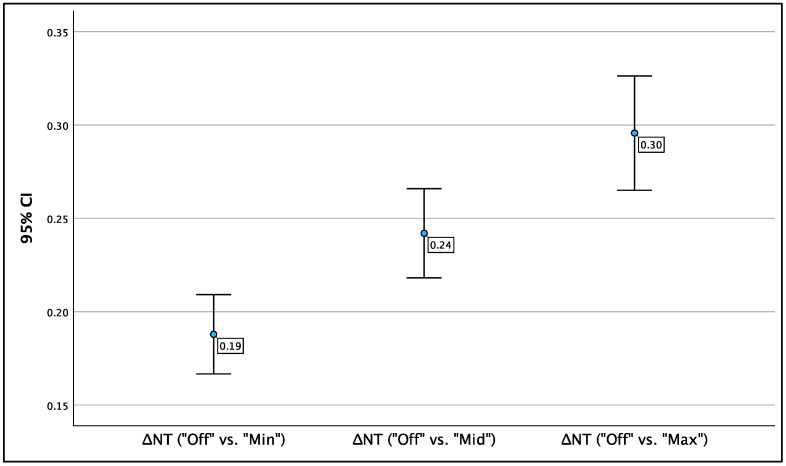
Mean ΔNT values, by mode of ‘Radiant’.

Comparing values without ‘Radiant’ applied to those with ‘Radiant‘ applied revealed a significant difference in average NT values. For clarity, this difference will be denoted as ‘ΔNT’. Significant discrepancies were observed across the three settings of ‘Radiant’—minimum, medium, and maximum—compared to ‘Radiant off’. Figure 5 illustrates the mean ΔNT values for each ‘Radiant’ method, with brackets indicating the respective 95% confidence intervals (95% CI).

The most notable difference in NT was observed between ‘Radiant off’ and ‘Radiant max’; on average, NT measurements were 0.30 mm greater when ‘Radiant max’ was applied. This difference was statistically significant (*p* < 0.001; 95% CI: 0.27–0.33). The ΔNT between ‘Radiant off’ and ‘Radiant min’ was smaller, averaging 0.19 mm, yet still significant (*p* < 0.001; 95% CI: 0.17–0.21). The average ΔNT between ‘Radiant off’ and ‘Radiant mid’ was 0.24 mm, also statistically significant (*p* < 0.001; 95% CI: 0.22–0.27). The results are presented in Table 1 and Figure 5.

### 3.2. Relation of Native NT and ΔNT

**Figure 6:**
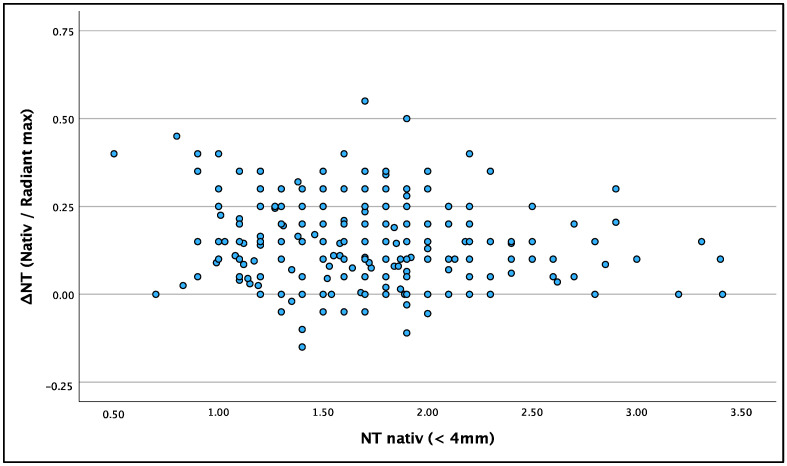
Correlation between Native NT and ΔNT (for ‘Radiant max’).

To examine if the radiant effect was more pronounced in large or small NT values, we examined whether ΔNT is dependent on the original NT value. There was no connection between native NT and ΔNT. Figure 6 shows ΔNT (for ‘Radiant max’) in relation to native NT values. The statistical correlation was poor with Fisher’s correlation at −0.152.

### 3.3. In Vitro Examination with and without ‘Radiant’ Applied

**Figure 7:**
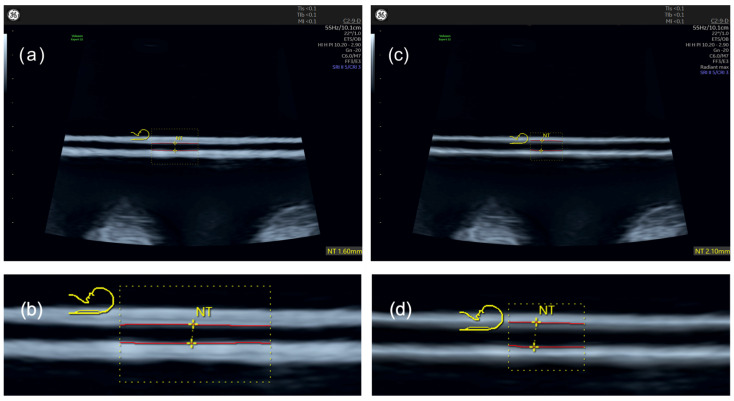
In vitro measurement of a 2.5 mm distance with and without ‘Radiant’. (**a**,**b**) Settings with ‘Harmonic Imaging’ and ‘Radiant off’, whole picture, and zoomed-in section below. (**c**,**d**) The same object with ‘Harmonic Imaging’ and ‘Radiant max’.

To corroborate our findings from live patient examinations, we established an in vitro setup with constant, predefined line distances of 1.0 and 2.5 mm, as previously described. The in vitro results align with those observed during live NT measurements. The ‘Radiant’ setting resulted in thinner, more defined line thicknesses. In the ‘Fundamental’ mode— the native B-image without enhancements—the lines were slightly thicker, with distances between lines increasing by 0.1 to 0.2 mm for both measured distances. ‘Harmonic Imaging’ did not affect smaller distances but had a more noticeable impact on larger ones, increasing line distance by up to 0.5 mm. With ‘Harmonic Imaging’, deviations from the actual distances were significant, showing a reduction of −0.3 mm for 1.0 mm of actual distance, and ranging from −0.4 to −0.9 mm for 2.5 mm of actual distance. This effect is visible in B-mode images as blurring and increased line thickness, which reduces the measured distance. The comparison of line thickness using only ‘Harmonic Imaging’ versus ‘Harmonic Imaging’ combined with ‘Radiant’ is depicted in Figure 7.

Using ‘Ultra HD’, measurements were generally more accurate, with a maximum deviation of −0.2 mm for both 1.0 mm and 2.5 mm of actual distances. ‘Ultra HD’ appears to enhance precision by reducing line thickness. Although the effect of combining ‘Radiant’ with ‘Ultra HD’ was observable, it was less dramatic than when combined with ‘Harmonic Imaging’. All in vitro measurement values are listed in Table 3 and Table 4.

Additionally, we observed that at smaller distances, measurements tended to increase up to ‘Radiant mid’ but decreased slightly at ‘Radiant max’. This pattern is documented in Table 3 for both ‘Fundamental’ and ‘Ultra HD’ settings. The trend was also evident during data acquisition in vivo, where smaller NT values initially increased with ‘Radiant mid’ but sometimes slightly decreased with ‘Radiant max’. However, this phenomenon was not evident in our in vitro measurement at a 2.5 mm line distance (see Table 4).

## 4. Discussion

This retrospective single-center cohort study was conducted to validate clinical observations and assess the impact of a novel image enhancement technology called ‘Radiant’. By applying ‘Radiant’ to in-device images post-examination, we simulated real patient scenarios, thereby creating a consistent experimental setting. To minimize confounding variables, each image was processed through all stages of ‘Radiant’. Additionally, we employed the ‘SonoNT’ tool to further enhance interobserver reliability.

Further, the ‘Radiant’ image enhancement technology significantly improves image quality by sharpening the visual output. This is evidenced by our controlled experiments, where the most accurate measurements of horizontal line distances were obtained using ‘Radiant mid’. Consequently, it appears that without the use of ‘Radiant’, NT measurements are consistently underestimated. In general, the measurement of horizontal line distances seems to be most precise when combining ‘Ultra HD’ and ‘Radiant’, because this combination created the least deviation from the actual distance presented (greyed in Table 3 and Table 4).

In our well-controlled in vitro setup, the measurement of horizontal line distances was most precise when combining ‘Ultra HD’ and ‘Radiant mid’, because this combination created the least deviation from the actual distance presented (greyed in Table 3 and Table 4). Without ‘Radiant’, all our in vitro measurements were underestimated. Consequently, one might propose that using ‘Radiant’ in vivo might reflect a more precise measurement.

When introducing a new diagnostic method that yields significantly different values, it is crucial to assess the clinical implications. Over a three-month period, our cohort of 263 cases demonstrated that the number of cases with nuchal translucency (NT) measurements exceeding 3.5 mm increased incrementally with each level of ‘Radiant’ enhancement applied. Specifically, without ‘Radiant’, four cases exceeded this threshold and were subjected to invasive testing (CVS or AC), confirming two cases of Trisomy 21, one of Trisomy 18, and one of Monosomy X. With ‘Radiant’ enhancements—‘Radiant min’, ‘Radiant mid’, and ‘Radiant max’—the numbers increased to five, six, and seven cases, respectively. Notably, the additional cases identified with ‘Radiant’ showed no further signs of aneuploidy in second-trimester screenings and did not require invasive procedures.

This results in a marked increase in the false-positive rate (FPR) from 0% with ‘Radiant off’ to 43% with ‘Radiant max’. However, drawing definitive conclusions from these rates is challenging due to the small sample size. Nonetheless, our in vitro results suggest that ‘Radiant’ may enhance the precision of NT measurements. The apparent contradiction between higher precision and increased FPR may indicate that the traditional reference values are outdated. Given that ‘Radiant’ tends to produce systematically larger, yet potentially more accurate, NT values, this discrepancy could be due to historically smaller reference values.

Recent technological advancements in ultrasound imaging underscore the need to revisit the NT reference tables established by Nicolaides et al. in 1992, which have been periodically updated in response to technological progress [1,8,12]. The ongoing debate about NT cutoff margins, including proposed thresholds of 3.0 mm, 3.5 mm, and the 99th percentile, should be informed by these advancements. These cutoffs are crucial as they align closely with current reference tables used in first-trimester screening [13].

To conclusively validate the findings reported, further investigations involving larger cohorts are essential. In practical terms, the use of ‘Radiant’ has subjectively enhanced the visual perception of anatomical landmarks in everyday clinical practice. This improvement not only supports the objective effects observed but also enhances the utility of this technology in routine first-trimester screening.

In our study, we observed that smaller NT values, around 1 mm, ‘Radiant max’ settings were slightly decreased compared to ‘Radiant mid’. This phenomenon was also replicated in our in vitro setup. However, the current dataset does not provide sufficient information to determine the cause of this effect.

Despite measures to minimize interobserver variations and create a consistent experimental setting, the generalizability of the study is limited due to its small sample size and the singular model of ultrasound device used, restricting the applicability of our findings to other settings or devices. At the time of manuscript preparation, no other device or brand offers technology comparable to ‘Radiant’.

For historical context, a similar enhancement in clinical practice was observed with the introduction of ‘Harmonic Imaging’ (HI) in the late 1990s. This technology markedly improved texture assessment and overall image quality, leading to its rapid integration as a standard feature in sonographic evaluations [14].

## 5. Conclusions

Given the observed increase in the precision of NT measurements and the enhanced overall image quality provided by ‘Radiant’, we recommend its adoption for first-trimester sonographic examinations. However, for clinical decision-making, we advise using ‘Radiant off’ mode until more comprehensive data on false-positive rates and updated NT reference values become available.

## Figures and Tables

**Table 1:** Average difference in NT values (ΔNT) between ‘Radiant off’ and each step of ‘Radiant’.

‘Radiant off’ vs.:	ΔNT	CI	SD	Significance
vs. ‘Radiant min’	0.19 mm	0.17–0.21	0.010	*p* < 0.001
vs. ‘Radiant mid’	0.24 mm	0.22–0.27	0.012	*p* < 0.001
vs. ‘Radiant max’	0.30 mm	0.27–0.33	0.016	*p* < 0.001

**Table 2:** Mean values by risk groups.

	Normal	Trisomy 21	Trisomy 18	Monosomy X
n	259 (98.48%)	1 (0.38%)	2 (0.78%)	1 (0.38%)
Mean GA	12w 4d	11w 6d	11w 6d	11w 2d
Mean CRL	64 mm	52 mm	51 mm	52 mm
Mean NT	1.98	6.97	6.36	7.10
Mean age	34	42	33	31
Mean BMI	25.7	24.9	20.4	26.0
NIPT rate *	61 (23.6%)	0	0	0
AC rate *	0	0	0	0
CVS rate *	4 (1.5%)	1 (100%)	2 (100%)	1 (100%)
ICSI/IVF *	17 (6.6%)	1 (100%)	0	0

* Absolute and relative numbers are given for NIPT (Non-Invasive Prenatal Testing), AC (Amniocentesis), CVS (Chorionic Villus Sampling), and assisted reproduction (ICSI/IVF: Intracytoplasmic Sperm Injection, In Vitro Fertilization) carried out in our study population.

**Table 3:** In vitro measurements of 1.0 mm distance (values in mm).

1.0 mm	Fundamental	HI High	Ultra HD
Radiant off	0.70	0.70	0.80
Radiant min.	0.90	0.70	0.90
Radiant mid.	0.90	0.70	1.00
Radiant max.	0.80	0.70	0.90

**Table 4:** In vitro measurements of 2.5 mm distance (values in mm).

2.5 mm	Fundamental	HI High	Ultra HD
Radiant off	2.20	1.60	2.30
Radiant min.	2.20	1.70	2.60
Radiant mid.	2.30	2.10	2.60
Radiant max.	2.40	2.10	2.60

## Data Availability

The research data from this study can be requested from the authors.
